# Health concerns about possible long-term effects of legally marketed milk and dairy from animals with intramammary infections

**DOI:** 10.3389/fpubh.2023.1200924

**Published:** 2023-08-28

**Authors:** Iris Schadt

**Affiliations:** Research Section for Nutraceuticals and Health Products, Consorzio per la Ricerca nel Settore della Filiera Lattiero-Casearia e dell'Agroalimentare (CoRFiLaC), Ragusa, Italy

**Keywords:** milk, mastitis, food safety, regulation, oxidation products, nitrosamines, cytokines, amyloid

## Abstract

Milk and dairy from animals with subclinical mastitis infections are marketable. Mastitis is detected with the somatic cell count (SCC). The EU regulation, among the stricter ones, limits an average of 400,000 somatic cells/ml in milk. Other countries have higher or no thresholds. This level suggests 40% of infected animals, and we indeed consume mastitic milk and dairy. A worldwide prevalence of dairy cattle and buffaloes with subclinical mastitis is estimated to range between 34 and 46%. The current food safety regulations account for mastitis pathogens, their toxins, and the risk of antimicrobial residues, but milk from animals with mastitis contains also compounds that derive from an immune response and inflammation process with biological function for the offspring. To the best of the current knowledge, it cannot be excluded that these compounds do not interfere with human homeostasis and that they do not contribute to redox or cytokine dysregulation that, in turn, could promote certain chronic diseases. These compounds include radicals, oxidation products, nitrosamines, and proinflammatory cytokines with nitrosamines being already recognized as probable carcinogens. Mastitis also alters the composition of caseins, plasmin, and plasminogen activators, which may be related to increased transformation into amyloid with similar characteristics as the fibrils associated with Alzheimer's disease. We should determine whether these bioactive compounds could, alone or in combination, represent any long-term risk to the consumer's health. Adapted regulations and concomitant subsidies for farmers are suggested, for sensing tools that reveal individual SCC and mastitis at milking. Frequent SCC determination is the prerequisite for any mastitis control program.

## 1. Background: bovine mastitis and current regulations for the commercialization of milk and dairy

Mastitis is an inflammation of the mammary gland generally associated with intramammary infection. The microorganisms primarily responsible for mastitis include bacteria such as *Escherichia coli, Staphylococcus aureus*, and *Streptococcus* spp. but fungi can also cause infection (1). The consumption of milk and dairy produced from animals with mastitis represents a worldwide recognized risk for public health, and many countries have adopted regulations for milk hygienic standards (2–5). These standards include maximum total bacteria counts, maximum counts of some distinct bacteria, and maximum somatic cell counts (SCC). Milk somatic cells are primarily leukocytes or white blood cells, which include macrophages, lymphocytes, and polymorphonuclear neutrophils (6). Epithelial cells contribute only to a low extent (2 to 15%) of the cell population. When bacteria enter the gland and establish an infection, inflammation is initiated accompanied by an influx of white cells from the bloodstream, altered secretory function, and changes in the volume and composition of secretion. Since cell numbers in milk are closely associated with inflammation and udder health, SCC is accepted as the international standard measurement of milk quality.

Even though being also affected by breed, environmental and physiological factors, the SCC is a much more accurate marker for mastitis compared to the traditional methods of microbial culturing that are known to provide a significant amount of false negative results (7). Depending on the country and state, actual legal limits for SCC vary between 400,000 and 1,000,000 cells/ml as follows (5): Brazil allows the highest maximum level of bulk tank SCC of 1,000,000 cells/ml. In the United States, the legal limit of bulk tank SCC for Grade A milk shipments is 750,000 cells/ml. At least two out of five consecutive samples collected that are tested 30–45 days apart need to be within this limit; otherwise, penalties will be imposed. A few US states have adopted lower limits ranging between 400,000 and 600,000 cells/ml of bulk tank SCC. Europe defines an upper limit of a rolling geometric average over 3 months of 400,000 cells/ml with at least one sample per month. Like Europe, also Australia, New Zealand, and Canada set the limit to 400,000 cells/ml.

## 2. Milk and dairy from cows with mastitis can be commercialized

The SCC of healthy quarters usually remains below 100,000 cells/ml (8, 9). However, under field conditions, to reduce the diagnostic error, an SCC below 200,000 cells/ml of milk is widely used as the threshold level to differentiate healthy udders from subclinically affected ones (10). This indicates that, currently, no country on the globe imposes legal restrictions preventing the commercialization of milk from cows with mastitis. Considering the best-case scenario of an upper limit of 400,000 cells/ml rolling geometric average over 3 months with one sample per month, it would be even able to commercialize milk with 800,000 cells/ml for an entire month, if the SCC could be then kept at 200,000 cells/ml in the two adjacent months. Values of bulk milk SCC of 100,000 to 200,000 cells/ml indicate that already ~20% of the animals in the herd are infected (11). Higher levels of 200,000 to 300,000 cells/ml, and 300,000 to 400,000 cells/ml, suggest 30% and 40% of infected animals, respectively.

## 3. Milk and dairy from cows with mastitis are indeed commercialized

According to the severity of the inflammation, mastitis can be classified in clinical or subclinical forms (12, 13). In clinical mastitis, besides the increased SCC, visible manifestations of infection are present (12, 13), such as abnormal milk (changes in color, presence of clots, and flakes), abnormal mammary gland (changes in tissue color and swelling), and changes in animal status (body temperature, appetite, and hydration level). The detection of clinical mastitis cases of course needs the appropriate attention of the farmer, but cases can be easily assessed including a pre-stripping step before milking evaluating the presence of clots. On the other hand, the SCC is the only measure to detect subclinical cases. In addition, subclinical mastitis occurs 15–40 times more often than the clinical form, and its duration is longer (14, 15). In this context, it is also to be noted that it is the milk from subclinical compared to clinical cases that have higher contents of pathogenic mastitis-causing bacteria (16).

A recent meta-analysis estimates the worldwide prevalence of dairy cattle and buffaloes with subclinical mastitis ranging between 34 and 46% (17). In particular, increasing prevalence estimates for subclinical mastitis of 34, 36, 37, 42, 44, 45, and 46% are reported for Latin America, Oceania, Europe, Asia, Africa, India, and North America, respectively. In China, considering the period 2012–2021, the prevalence of subclinical mastitis in dairy cows was 37.7% on average but varied between individual regions and reached the highest level of 72% in the Inner Mongolia Autonomous Region (18). The milk of these animals is part of the commercialized bulk milk as long as legal standards are met. Within a control program to manage mastitis *from Streptococcus agalactiae* infections, carried out in the Emilia-Romagna Region, in Italy, during 2019–2021, 17,056 bulk tank milk samples from 2,831 dairy herds were evaluated for SCC and the presence of *Streptococcus agalactiae* (19). Within these samples, there were marketable samples with an average SCC of 324,526 cells/ml, which were *Streptococcus agalactiae* positive. This suggests that the presence of *Streptococcus agalactiae* in marketable milk is a reality. When *Streptococcus agalactiae* was not present, the average SCC was still higher than 200,000 cells/ml, suggesting infections from other pathogens.

We are consuming milk and dairy from animals with mastitis, and the percentage of infected animals that contribute to that milk and dairy can be quite significant. Industry and research are of course aware of that fact. The threshold of ~200,000 cells/ml or less to distinguish uninfected from infected animals is known since the late 1970's (20). To comply with legal limits, the industry adopts mastitis control programs that use this threshold (21), and these programs are developed and promoted by researchers and research institutions (21–23). However, consumers may not know that marketable milk could be from animals with subclinical mastitis, because this information is simply not distributed to the public. Current food safety regulations may not fully account for possible biohazards from the consumption of this milk and dairy, especially those hazards that could be related rather to longer-term conditions and chronic diseases.

## 4. Four good reasons why the commercialization of milk from cows with mastitis should be of concern for public health

(1) Milk from animals with mastitis may contain pathogens, and there is a risk of the transmission of zoonoses. *Staphylococcus aureus* is not only among the major pathogens responsible for subclinical mastitis but also is known as one of the most important foodborne pathogens. In Europe, milk and dairy products account for 5% of all the incriminated foods in staphylococcal outbreaks (24). Mastitis pathogens play a minor role in drinking milk that is usually heat treated but can represent an issue in raw milk cheeses, in particular fresh cheeses (25). In addition, there is a risk that these pathogens could consist of antimicrobial-resistant strains (26). (2) Milk from animals with mastitis could contain toxins deriving from these pathogens and (3) may contain antimicrobial residues. Antimicrobial residues are only suspected when milk derives from animals with clinical mastitis, as subclinical cases are rarely treated with antibiotics. If present, both toxins and antimicrobial residues may represent harm independently of milk heat treatment. To some extent, these three potential hazards are considered by the current safety regulations by defining threshold limits. However, as has been already mentioned, pathogens are likely to be present in milk from subclinical animals but may not necessarily be detected.

The current regulations do not consider the presence or altered levels of other chemical and biological compounds that are also present in milk from animals with mastitis, compounds that derive from an immune response and inflammation process. (4) As such, in milk, these compounds have a biological and physiological function to protect the offspring. Analog compounds are produced also in the human body to regulate its homeostasis. The contents of most of these ingredients in milk and dairy are still not well known, and there is only very limited or no knowledge of their possible bioactivity after consumption and interference with human homeostasis. However, the absence of such knowledge does not necessarily preclude that these products do not pose any harm to human health. They could represent biohazards, either alone or in combination with each other. The following text is intended to illustrate the current knowledge about these compounds in milk and dairy associated with mastitis and to justify the concerns about possible health implications. Such chemical and biological compounds include the following:

### 4.1. Free radicals, oxidation products, and nitrosamines

Infection and inflammation involve altered redox balance and induce oxidative stress (27). Mastitis is associated with the release of free radicals, increased total oxidant capacity, and decreased total antioxidant capacity in milk (28–31). As part of the immune response to udder infection, reactive oxygen species (ROS) are released by immune cells and play a vital role in combating pathogens. Under normal physiological conditions, ROS production and clearance are in dynamic equilibrium, and ROS can be maintained at extremely low beneficial and harmless levels (32). However, an imbalance in the redox status of the individual animal due to excessive ROS production or impaired antioxidant defense can result in oxidative stress. Cows with mastitis compared to healthy cows appear to have approximately a 10-fold oxidative stress index (33). ROS are highly reactive, including hydrogen peroxide, singlet oxygen, and radicals and can interact with lipids and proteins. Increased ROS levels not only decrease milk quality (28, 34, 35) but could also pose a risk to the consumer (36). Lipid oxidation in food produces as primary products lipid hydroperoxides, which then decompose into a variety of oxygenated and aliphatic fatty acid scission products (37). Lipid hydroperoxides are unstable and do not seem to be absorbed into the plasma. Research on the toxicity of lipid oxidation products in foods has therefore focused on secondary lipid oxidation products (37). Specific examples of lipid oxidation compounds in food that are of health concern include malonaldehyde (MDA) and several cholesterol oxidation products (37–39). As judged from the results of animal experiments, the involvement of oxysterols in atherogenesis is highly controversial because they are also formed in the body and are also involved in some physiological processes but cannot be excluded (40). Cholesterol is up to 10 times less susceptible to oxidation compared to polyunsaturated fatty acids (37).

MDA of approximately 20, 30, and 55 nmol/ml in milk from healthy cows, compared to cows with subclinical and clinical mastitis, respectively, was reported (41, 42). In another study, MDA values were 16 and 46 nmol/ml in milk from either healthy cows or cows with subclinical mastitis (43) suggesting up to triplicate MDA contents when milk is obtained from animals with udder infections. Milk lipid oxidation and MDA contents are also affected by the level of production (44). Milk from healthy cows in their second lactation, at peak lactation, had increasing MDA levels of approximately 20, 40, and 60 nmol/ml at daily milk production levels of 37, 45–49, and 50–56 kg, respectively. This indicates that milk MDA levels at higher production levels with mastitis could be further altered compared to non-mastitis conditions. Subclinical mastitis reduces milk production (45, 46), but higher production levels still may be reached. Mastitis milk contains higher cholesterol levels (47) suggesting also higher contents of cholesterol oxidation products. As mentioned before, also increased protein oxidation may occur with udder infections. However, so far, specific measurements of cholesterol, protein, or other lipid oxidation products in dairy products associated with mastitis are not reported in the literature.

Radicals, other ROS, and cholesterol oxidation products have also endogenous origin, can have physiological functions, and are combated with antioxidants of both exogenous and endogenous origin, to maintain the body in a balanced redox status (32, 40, 48–50), and the oral toxicity of oxidized lipids seems to be low (51). Various food categories including full-fat milk and cheese were analyzed for MDA and two other lipid oxidation products in combination with consumption data obtained from a national representative sample of the Belgian population, for quantitative exposure, and risk assessment (52) applying the Threshold of Toxicological Concern (TTC) concept for chemicals with no toxicity data available. Based on the Cramer decision tree, MDA was classified into class I considering a TTC level of exposure of 30 μg/kg body weight per day. Under this condition, only a small proportion (3.8%) of those who consume cured and minced raw meat could be at risk. According to that study, the consumption of milk and cheese, however, does not appear to pose a risk. Higher MDA levels in milk and cheese due to udder infections were not considered in that study but would not probably make a difference in the possible outcomes. Nonetheless, to prevent premature conclusions, various aspects should also be considered. Milk and dairy products are part of a diet and as such mastitis infections and altered MDA contents still could make a difference in whether the entire diet is healthy or not, even if these foods didn't represent a risk as individual ingredients. Furthermore, this analysis assesses only the exposure risk of individual chemical compounds. In the case of mastitis milk and dairy, there are various altered compounds with potential biological activity. Even if these compounds had no affirmed individual effect, their combined presence still could have an effect. Moreover, limitations of the TTC principle include a lack of specificity not accounting for specific endpoints or individual susceptibility. In addition, dietary lipid oxidation products could have a greater impact on individuals with underlying health conditions and, as pointed out below, appear to also target specific organs. Finally, the same authors point out that no robust toxicological data are available yet and that therefore, regardless of the study outcomes, precautions should be taken to prevent lipid oxidation in foods.

Chronic uptake of large amounts of lipid oxidation products increased tumor frequency and incidence of atherosclerosis in animals (51, 53–55). Especially in poorly controlled type 2 diabetic patients, but also in healthy subjects, oxidized cholesterol in the diet was found to be a source of oxidized lipoproteins in human serum (56–58) suggesting that these increased postprandial levels of potentially atherogenic oxidized lipids may contribute to the accelerated atherosclerosis associated with diabetes. Some of the dietary lipid oxidation products, which are absorbed from the gut to the circulatory system, seem to activate an inflammation response that affects not only the circulatory system but also specific organs such as the liver, kidney, lung, and the gut itself (39). As for protein oxidation products, again, the formation of these products would not only compromise milk and dairy quality but may also affect food safety (59). When rats were fed diets containing milk powder varying in protein oxidation state, rats responded with respectively varying degrees of redox state imbalance and oxidative damage in plasma, liver, and brain tissues. Furthermore, hippocampal inflammatory and apoptosis genes were significantly upregulated in groups with higher oxidation states, while learning and memory genes were significantly downregulated (59).

Besides ROS, also reactive nitrogen species are involved in the inflammatory response during mastitis. Nitric oxide-derived oxidative stress caused by mastitis of cows and goats increases milk nitrate (28, 35), nitrite (28, 60), nitric oxide (NO) (28, 35), and S-Nitrosamines (28). Cows with mastitis compared to healthy cows appear to have about doubled plasma and milk NO levels (33, 61). In the acidic environment of the stomach, nitrate can be reduced to nitrite and nitrite converts to NO and nitrosamine compounds. Nitrosamine compounds are formed by the reaction of nitrosatable amines (62). Within these compounds, it may be the nitrosamines that are concerning (63). The International Agency for Research on Cancer (IARC) has considered nitrosamine compounds as probable carcinogens for humans (group 2A) (64). A health risk assessment of nitrosamines was conducted as a pilot study in Iran where 33 healthy adults participated in and completed a food frequency questionnaire for 3 days. According to that survey, and literature data on the concentrations of these compounds in the various food categories, dairy products seemed to have a significant impact on the daily intake of nitrosamines. The highest contributor to the daily intake of nitrosamines was meat, followed by dairy products that corresponded to about a third of that provided by meat. Fruits and grains had a minor impact. The estimated cancer risk from the dietary intake of nitrosamines ranged between 1.74 and 2.22 × 10^−3^ with a 95% confidence interval (62). In this context, mastitis can be a game changer. In goat milk from uninfected udders, S-nitrosamines concentrations were only 0.17 (μM) but increased ~14-fold to 2.4 (μM) in milk from infected udders (28).

### 4.2. Cytokines

Cytokines are peptides that have critical roles in immunology and include interleukins (IL), interferons (IFN), lymphokines, chemokines, tumor necrosis factors (TNF), and transforming growth factors (TGF). They are involved in immune response, health status, and tissue development and can act as tissue hormones in an autocrine and paracrine fashion. Cytokines serve as either proinflammatory or anti-inflammatory agents; on the one hand, as neutralizers of harmful pathogenic effects and on the other hand, as inducers for the maturation and development of the immune system (65). Major depression (66), Alzheimer's disease (67), cancer (68), and autoimmune diseases (69) are accompanied by an inflammatory response. However, it appears that increased levels of proinflammatory cytokines are not just symptoms of these diseases. Recent evidence also indicates that a dysfunctional neuroendocrine-immune interface associated with abnormalities of the “systemic anti-inflammatory feedback” and/or “hyperactivity” of the local proinflammatory factors may play a role in the pathogenesis of atopic/allergic and autoimmune diseases, obesity, depression, and atherosclerosis (70, 71). There are also various studies that prove the role of inflammation in the genesis and proliferation of certain tumors such as lung cancer (72), colitis-associated colorectal cancer, and cholangiocarcinoma (73). It appears from a recent study that used information from a lifestyle questionnaire obtained from 150 healthy individuals and their serum samples that red meat consumption seems to be associated with an inflammatory pattern, characterized by an increase in IL-6 and IL-8. IL-8 levels were also increased with the frequent intake of sweets, while a higher intake of shelled fruits correlated with lower levels of IL-6 (74). High body mass index had a higher impact on the serum levels of C-reactive protein than the consumption of individual food categories and, in that context, the consumption of milk and dairy had no influence. However, the study outcomes may not apply to individuals with pre-existing medical conditions. In addition, this study did not account for possible variations in udder infection status or SCC. As shown in the following paragraph, milk cytokine levels are altered with mastitis. Theoretically, the consumption of foods could contribute to cytokine dysregulation or inflammation not only by stimulating the production of proinflammatory cytokines in the body but also by providing these cytokines directly through the foods.

The leukocytes that come with milk from infected udders are involved in the production of milk cytokines (75). Cytokines are also present in milk from healthy udders, but composition and concentrations change with both clinical and subclinical mastitis (16) depending on time after exposure, pathogen species, and severity of the disease (76). Proinflammatory cytokines as indicators of early inflammation such as TNF-α appear to reach a peak in 1–12 h after exposure followed by a gradual drop (77), whereas other cytokines can reach their maximum levels at up to 7 days or even later (76) depending again also on the pathogen species. In naturally acquired mastitis, milk concentrations of IFN-γ reached up to 20 ng/ml, whereas maximum reported levels of IL-6, TNF-α, IL-1β, IL-8, and TGF-α were about 90 ng/ml, 25 ng/ml, 8 ng/ml, 1 ng/ml, and 0.5 ng/ml, respectively, values that were significantly higher than in milk from healthy udders (76). In comparison, the control milk samples had maximum levels of about only 0–0.15 ng/ml of IFN-γ, TNF-α, IL-1β, IL-8, and TGF-α (76).

Milk cytokines are crucial for the development of the newborn's organism and its immune system (78). Cytokines have been also considered for therapeutic use, and in that context, it has been demonstrated that orally administered interferons and cytokines can exert both local and systemic effects (79). TGF-β, for example, when synthesized as an inactive precursor, can be activated during intestinal transit by multiple mechanisms, e.g., by a low pH of 1.5 (65). It is therefore probable that milk cytokines are bioactive after ingestion and digestion. Cytokines have very complex biological functions and can interact with each other. Some can induce the production of others, act synergistically to enhance their effects, inhibit the expression of others, or stimulate the expression of receptor antagonists or receptors of others (76). For example, IL-1 induces the production of IL-1 itself, TNF-α, IL-6, IL-8, and IL-12. In the cases of TNF-α and IL-6, IL-1 can act synergistically to enhance their effects (76). This suggests that cytokines in food could make a difference even if present in apparently small amounts. Almost 30 different cytokines with specific features (65) have been already identified in milk raising the question about their biological immunomodulatory significance as well as their risk for human consumption, which has still to be determined.

### 4.3. AA amyloid

Serum amyloid A (SAA) is a precursor in the formation of AA fibrils which, in turn, are associated with various, especially neurodegenerative, diseases, such as Alzheimer's. It has been demonstrated in rodent models that AA fibrils can be orally transmitted (80). The AA fibrils cross the gut barrier (81) and can trigger the disease. The oral “infectious dose” of AA fibrils, at <1 μg, is comparable with the infectivity of prions (82), and species barriers can be surmounted (81). Also, cooking temperatures are unable to eliminate their amyloidogenic potential (82). Presumably because of similarities in structure and composition, AA fibrils exhibit similar resistance to physical and chemical decontamination methods. Treatment with cooking (82), freezing/thawing, and disinfectants such as formalin and 2N NaOH were not able to abolish AA fibril infectivity. Not even autoclaving for 3 h could guarantee inactivation (82). Transgenic mice that were fed extracts of fibrillar material composed of serum amyloid from *fois gras* developed amyloid deposits in virtually all organs examined (80). The authors concluded that an amyloid-containing food product hastened the development of amyloid protein A amyloidosis in a susceptible population. However, AA amyloidosis seems to only develop in the concomitant presence of high levels of SAA with AA-derived fibril seeds (83). Healthy mice exposed orally to AA fibrils did not develop amyloidosis, whereas those additionally receiving a concurrent inflammatory stimulus developed a pronounced disease within days (83). Conditions associated with elevated SAA levels may include chronic infections, as well as non-infectious chronic inflammatory diseases, and certain tumors (84). The concentration of SAA can drastically increase at an acute inflammation as a response to cytokines, especially IL-1, IL-6, and TNF-α (85). On the other hand, it also has been shown that even months after fibril exposure, an inflammatory stimulus could rapidly induce AA amyloidosis to the same extent as concurrent inflammation and fibril injection (83). In mice, the longest interval between exposure and inflammation studied was 180 days, which corresponds to nearly one-quarter of the animals' natural life span (83). This would suggest that consumers of food with high levels of AA fibrils may be at increased risk for AA amyloidosis in case they developed an inflammatory disorder even years after consumption.

As discussed before, the milk from animals with mastitis would provide cytokines IL-1, IL-6, and TNF-α. In addition, in response to mastitis inflammation, higher levels of milk AA are expected (86–88), levels depending on the pathogen (87). Compared to pathogen-free milk with an AA concentration of approximately 12 μg/ml, AA levels in milk from animals with subclinical mastitis from *Staphylococcus aureus, Streptococcus agalactiae, Streptococcus dysgalactiae, Streptococcus uberis*, and *Candida* spp. were ~42, 60, 72, 92, and 102 μg/ml, respectively (87). Milk from animals with coagulase-negative staphylococci subclinical infections instead was not different from the pathogen-free milk. The AA fibril content in milk and dairy from animals with mastitis may be elevated, considering an altered casein composition. Amyloid fibril appears to be formed by αs2- and κ-casein and seems to be inhibited by αs1- and ß-casein (89). The overall casein concentration in milk from animals with mastitis seems to be reduced (90, 91), especially that of β- (92, 93) and αs1-casein (92). At the same time, the contents of κ-casein may be increased (93). In addition to the altered casein composition, milk from infected udders also exhibits greatly increased proteolytic activity (90, 94, 95). Both, plasmin and plasminogen activators are increased when milk is from infected udders (96–98). Moreover, a higher pH in milk from animals with mastitis (90, 99) favors the plasminogen activators (100). Plasminogen activation and plasmin activity appear to be involved in transthyretin amyloidosis including the initiation, progression, and tissue distribution of amyloid deposition (101, 102).

Recently, a concern has been raised that AA amyloid in the human food chain could represent a possible biohazard (103). On that behalf, it was investigated whether amyloid seeds from different food proteins (lysozyme, β-lactoglobulin, soybean, mung bean, fava bean, lupine, potato, oat) affected the kinetics of Aβ_1−42_ amyloid formation, which is the particular variant associated with Alzheimer's disease (104). None of the tested seeds had fibrillation-promoting effects. However, the authors noted differences between the protein sources. Lysozyme, for example, had even an inhibiting effect on Aβ_1−42_ fibrillation. This suggests that the results of this study may not be exhaustive and may not represent all food proteins. Other food proteins, that have not yet been tested, still could promote fibrillation. Amyloid fibril formation by casein and fatty acids was observed in the human breast milk of mastitis patients (105).

### 4.4. Cortisol

In addition to the abovementioned compounds, intramammary infections also increase milk cortisol levels (106). Milk cortisol levels with SCC of <200,000 cells/ml, between 200,000 and 400,000 cells/ml, and of >400,000 cells/ml were 470, 470, and 530 pg/ml, respectively (106). However, this difference appears to be small compared to the cortisol levels that are already present in milk from healthy udders, suggesting that mastitis milk could only worsen to some extent a possible persistent risk. Fludrocortisone, which has over a 100-fold mineralocorticoid potency compared to hydrocortisone, is used to treat adrenocortical deficiency and orthostatic hypotension, and daily doses of 0.1 to 0.2 mg are considered to have only very little or no glucocorticoid effect at all (107). It is rather unlikely to possibly reach similar levels of cortisol from the consumption of milk and dairy products alone but we might also keep in mind that cortisol is a hydrophobic molecule, that should be more concentrated in whole-milk cheese and cream than in milk, and that cortisol may also be present in other animal-derived foods.

## 5. Recommendations for governments and food safety authorities: research, regulations, and support

### 5.1. Research and regulations

Governments and food safety authorities should support and commission research to assess whether any of these bioactive compounds could, considering also their combination, represent any risk to the consumer's health considering also possible long-term effects. We might then also need to further investigate the possible concentrations of these compounds in milk from cows with mastitis. This information would be necessary for the legislators to either confirm that the current regulations are sufficient to guarantee the safety of milk and dairy products or to eventually adapt the SCC thresholds for the commercialization of milk and dairy. However, in any case, when it comes to products with any type of quality label, more restrictive regulations concerning milk hygiene should be considered. These labels not only give assurance about the traceability of the products to specific areas of production and/or the application of specific sets of competencies and know-how, but the consumer may also associate this assurance with distinctively high quality, in general, as well as high sanitary quality and safety standards, in particular, even if these standards are not taken into account.

### 5.2. Support for the dairy industry

To reach the goal of milk from mastitis-free animals, regulations alone cannot be sufficient, but financial support for farmers may be necessary. In Europe, mastitis control has become even more difficult since 28 January 2022, when the blanket antibiotic dry cow therapy has been prohibited under Regulation (EU) 2019/6 on veterinary medicinal products. This restriction ought to reduce on-farm antibiotic use to reinforce the EU's strategy against antimicrobial resistance. Selective dry cow therapy is suggested as an alternative instead. However, many farmers will need training, and the latter practice may require also higher attention from the farmer and more specific testing of the individual animals that are to be dried off. Selective dry cow therapy compared to blanket dry cow treatment may increase SCC after calving, especially when cows at dry-off are not treated with an internal teat sealant (108, 109). It is in particular the small-and medium-sized farms that will need subsidies. There are several management practices and protocols to contempt and prevent mastitis, but as a prerequisite, first of all, the animals with mastitis need to be detected. Therefore, frequent individual SCC measurements are obligatory. The best solution to this problem would be the investment in sensing tools that are incorporated into the milking system. In many modern farms of a certain size, such tools are already part of automated milking systems and robots, capable of providing online measurements and immediate alerts to the farmers. Many of the smaller farms instead, where the economic sustainability of dairy farming is at risk, may not be able to afford such sensors, and subsidies should be tailored especially for those situations. With this regard, it should be kept in mind that very often Common Agricultural Policy's subsidies are more likely to reach the larger farms (110). Another economic aid could be provided in certain circumstances for the culling of animals with ascertained specific contagious intramammary infections when it is unlikely that antibiotic treatments can eradicate the infection. Antibiotic treatments of animals with chronic *Staphylococcus aureus* infections, for example, have a very reduced chance of only 35% to be efficient (111), and increase the development of antibiotic-resistant strains at the same time (112). Animals with contagious mastitis infections should be segregated from healthy animals in the milking parlor, but in the longer term, this practice cannot be able to eliminate the risk of spreading the infections. On the other hand, for the farmer, the decision to cull these animals is often very difficult, especially when large parts of the herd are involved, and the number of replacement heifers is not sufficient to maintain the herd size at a certain level.

## Data availability statement

The original contributions presented in the study are included in the article/supplementary material, further inquiries can be directed to the corresponding author.

## Author contributions

The author confirms being the sole contributor of this work and has approved it for publication.

## References

[1] Haxhiaj K Wishart DS Ametaj BN. Mastitis: what it is, current diagnostics, and the potential of metabolomics to identify new predictive biomarkers. Dairy. (2022) 3:722–46. 10.3390/dairy3040050

[2] European Union. Regulation (EC) No 852/2004 of the European Parliament and of the Council of 29 April 2004—On the hygiene of foodstuffs. Off J. (2004) 139:1–54. Available online at: https://eur-lex.europa.eu/legal-content/EN/TXT/PDF/?uri=CELEX:32004R0852/ (accessed May 3, 2023).

[3] European Union. Regulation (EC) No 853/2004 of the European Parliament and of the Council of 29 April 2004—Laying down specific hygiene rules for food of animal origin. Off J. (2004) 139:55–205. Available online at: https://eur-lex.europa.eu/legal-content/EN/TXT/PDF/?uri=CELEX:32004R0853/ (accessed May 3, 2023).

[4] European Union. Regulation (EC) No 1441/2007 amending Regulation (EC) No 2073/2005 on microbiological criteria for foodstuffs. Off. J. (2007) 322:12-29. Available online at: https://eur-lex.europa.eu/legal-content/EN/TXT/PDF/?uri=CELEX:32007R1441/ (accessed May 3, 2023).

[5] Animal Plant Health Inspection Service U.S. Department of Agriculture. Determining U.S. Milk Quality Using Bulk-Tank Somatic Cell Counts, 2016. [Fact sheet]. (2018). Available online at: https://www.aphis.usda.gov/animal_health/nahms/dairy/downloads/dairy_monitoring/BTSCC_2016infosheet.pdf/ (accessed March 10, 2023).

[6] Li N Richoux R Boutinaud M Martin P Gagnaire V. Role of somatic cells on dairy processes and products: a review. Dairy Sci Technol. (2014) 94:517–38. 10.1007/s13594-014-0176-3 25309683 PMC4180028

[7] Ashraf A Imran M. Diagnosis of bovine mastitis: from laboratory to farm. Trop Anim Health Prod. (2018) 50:1193–202. 10.1007/s11250-018-1629-0 29948774

[8] Hamann J. Mastitis in Dairy Production: Current Knowledge and Future Solutions. Proc. 4th International Mastitis Seminar, IDF, Mastitis in Dairy Production, Current Knowledge and Future Solutions, Wageningen Acad, Publ., Wageningen, the Netherlands. (2005) pp. 82–90. 10.3920/978-90-8686-550-5

[9] Sumon SMMR Parvin MS Ehsan MA Islam MT. Relationship between somatic cell counts and subclinical mastitis in lactating dairy cows. Veterinary World. (2020) 13:1709–13. 10.14202/vetworld.2020.1709-1713 33061248 PMC7522932

[10] Petzer IM Karzis J Donkin EF Webb EC Etter E. Somatic cell count thresholds in composite and quarter milk samples as indicator of bovine intramammary infection status. Onderstepoort J Vet Res. (2017) 84:1–10. 10.4102/ojvr.v84i1.1269 28397516 PMC6238690

[11] O'Brien B. Moorepark dairy levy research update no. 8. Practical steps to improve milk quality. In: *Milk Quality Handbook*. Cork: Teagasc, Moorepark Food Research Centre (2008). Available online at: https://t-stor.teagasc.ie/handle/11019/866/ (accessed August 13, 2023).

[12] Leitner G Lavon Y Merin U Jacoby S Blum SE Krifucks O. Milk quality and milk transformation parameters from infected mammary glands depends on the infecting bacteria species. PLoS ONE. (2019) 14:e0213817. 10.1371/journal.pone.0213817 31260459 PMC6602173

[13] Cheng WN Han SG. Bovine mastitis: risk factors, therapeutic strategies, and alternative treatments—A review. Asian-Australas J Anim Sci. (2020) 33:1699–713. 10.5713/ajas.20.0156 32777908 PMC7649072

[14] Shearer JK Harris B Jr. Mastitis in Dairy Goats. Gainesville, USA: The University of Florida, Institute of Food Agricultural Sciences, Extension. (2003). Available online at: http://ufdcimages.uflib.ufl.edu/IR/00/00/47/55/00001/DS12000.pdf/ (accessed March 10, 2023).

[15] Seegers H Fourichon C Beaudeau F. Production effects related to mastitis and mastitis economics in dairy cattle herds. Vet Res. (2003) 34:475–91. 10.1051/vetres:2003027 14556691

[16] Vitenberga-Verza Z Pilmane M Šerstnova K Melderis I Gontar Ł Kochański M. Identification of inflammatory and regulatory cytokines IL-1α-, IL-4-, IL-6-, IL-12-, IL-13-, IL-17A-, TNF-α-, and IFN-γ-producing cells in the milk of dairy cows with subclinical and clinical mastitis. Pathogens. (2022) 11:372. 10.3390/pathogens11030372 35335696 PMC8954094

[17] Krishnamoorthy P Goudar AL Suresh KP Roy P. Global and countrywide prevalence of subclinical and clinical mastitis in dairy cattle and buffaloes by systematic review and meta-analysis. Res Vet Sci. (2021) 136:561–86. 10.1016/j.rvsc.2021.04.021 33892366

[18] Chen X Chen Y Zhang W Chen S Wen X Ran X. Prevalence of subclinical mastitis among dairy cattle and associated risks factors in China during 2012-2021: a systematic review and meta-analysis. Res Vet Sci. (2022) 148:65–73. 10.1016/j.rvsc.2022.04.007 35513909

[19] Tamba M Rocca R Prosperi A Pupillo G Bassi P Galletti G. Evaluation of control program against streptococcus agalactiae infection in dairy herds during 2019–2021 in Emilia-Romagna Region, Northern Italy. Front Vet Sci. (2022) 9:904527. 10.3389/fvets.2022.904527 35812887 PMC9261462

[20] Eberhart RJ Gilmore HC Hutchinson LJ Spencer SB. Somatic cell counts in DHI samples. Proc Natl Mastitis Counc. (1979) 32:4.

[21] Nickerson SC Oliver SP. REVIEW: how well have United States dairy producers adopted mastitis-control technologies for reducing herd somatic cell counts improving milk quality? Profess Animal Sci. (2014) 30:115–24. 10.15232/S1080-7446(15)30098-X

[22] Lam TJ van den Borne BH Jansen J Huijps K van Veersen JC van Schaik G. Improving bovine udder health: a national mastitis control program in the Netherlands. J Dairy Sci. (2013) 96:1301–11. 10.3168/jds.2012-5958 23245961

[23] Østerås O Sølverød L. Norwegian mastitis control programme. Ir Vet J. (2009) 62:S26. 10.1186/2046-0481-62-S4-S26 22081877 PMC3339347

[24] Bianchi DM Gallina S Bellio A Chiesa F Civera T Decastelli L. Enterotoxin gene profiles of Staphylococcus aureus isolated from milk and dairy products in Italy. Lett Appl Microbiol. (2014) 58:190–6. 10.1111/lam.12182 24151939

[25] Gajewska J Zakrzewski A Chajecka-Wierzchowska W Zadernowska A. Meta-analysis of the global occurrence of S. *aureus* in raw cattle milk and artisanal cheeses. Food Control. (2023) 147:109603. 10.1016/j.foodcont.2023.109603

[26] Caruso M Latorre L Santagada G Fraccalvieri R Miccolupo A Sottili R. Methicillin-resistant Staphylococcus aureus (MRSA) in sheep and goat bulk tank milk from Southern Italy. Small Ruminant Research. (2016) 135:26–31. 10.1016/j.smallrumres.2015.12.023 27217357

[27] Lykkesfeldt J Svendsen O. Oxidants and antioxidants in disease: oxidative stress in farm animals. Vet J. (2007) 173:502–11. 10.1016/j.tvjl.2006.06.005 16914330

[28] Silanikove N Merin U Shapiro F Leitner G. Subclinical mastitis in goats is associated with upregulation of nitric oxide-derived oxidative stress that causes reduction of milk antioxidative properties and impairment of its quality. J Dairy Sci. (2014) 97:3449–55. 10.3168/jds.2013-7334 24704229

[29] Yang FL Li XS. Role of antioxidant vitamins and trace elements in mastitis in dairy cows. J Adv Vet Anim Res. (2015) 2:1–9. 10.5455/javar.2015.b4818325801

[30] Kleczkowski M Kluciński W Shaktur A Sikora J. Concentration of ascorbic acid in the blood of cows with subclinical mastitis. Pol J Vet Sci. (2005) 8:121–5. 15989131

[31] Ranjan R Swarup D Naresh R Patra RC. Enhanced erythrocytic lipid peroxides and reduced plasma ascorbic acid, and alteration in blood trace elements level in dairy cows with mastitis. Vet Res Commun. (2005) 29:27–34. 10.1023/b:verc.0000046740.59694.5d 15727289

[32] Ludin A Gur-Cohen S Golan K Kaufmann KB Itkin T Medaglia C. Reactive oxygen species regulate hematopoietic stem cell self-renewal, migration and development, as well as their bone marrow microenvironment. Antioxid Redox Signal. (2014) 21:1605–19. 10.1089/ars.2014.5941 24762207 PMC4175025

[33] Mohamed Ibrahim HM El-seedy YY Gomaa NA. Cytokine response and oxidative stress status in dairy cows with acute clinical mastitis. J Dairy Vet Anim Res. (2016) 3:00064. 10.15406/jdvar.2016.03.00064

[34] Paixão MG Abreu LR Richert R Ruegg PL. Milk composition and health status from mammary gland quarters adjacent to glands affected with naturally occurring clinical mastitis. J Dairy Sci. (2017) 100:7522–33. 10.3168/jds.2017-12547 28647336

[35] Novac CS Andrei S. The impact of mastitis on the biochemical parameters, oxidative and nitrosative stress markers in goat's milk: a review. Pathogens. (2020) 9:882. 10.3390/pathogens9110882 33114454 PMC7693667

[36] Vargas RT Dias Andrade PV Pinho Cerqueira MMO Nogueira Souza F Ribeiro Guimarães J Guimarães Ladeira CV. Influence of reactive oxygen and nitrogen species on udder health and milk quality. Rev Inst Laticí*nios Cândido Tostes, Juiz de Fora*. (2021) 76:131–41. 10.14295/2238-6416.v76i2.851

[37] Vieira SA Zhang G Decker EA. Biological implications of lipid oxidation products. J Am Oil Chemists' Soc. (2017) 94:339–51. 10.1007/s11746-017-2958-2

[38] Addis PB. Occurrence of lipid oxidation products in foods. Food Chem Toxicol. (1986) 24:1021–30. 10.1016/0278-6915(86)90283-8 3542756

[39] Kanner J. Dietary advanced lipid oxidation endproducts are risk factors to human health. Mol Nutr Food Res. (2007) 51:1094–101. 10.1002/mnfr.200600303 17854006

[40] Vejux A Malvitte L Lizard G Side. effects of oxysterols: cytotoxicity, oxidation, inflammation, and phospholipidosis. Braz J Med Biol Res. (2008) 41:545–56. 10.1590/s0100-879x2008000700001 18719735

[41] Zigo F Elecko J Vasil M Ondrasovicova S Farkasova Z Malova J. The occurrence of mastitis and its effect on the milk malondialdehyde concentrations and blood enzymatic antioxidants in dairy cows. Vet Med. (2019) 64:423–32. 10.17221/67/2019-VETMED

[42] Yang FL Li XS He BX Yang ZL Li GH Liu P. Malondialdehyde level and some enzymatic activities in subclinical mastitis milk. Afr J Biotechnol. (2011) 10:5534–8. 10.5897/AJB10.2226

[43] Yehia SG Shawky Ramadan E Saad M Mosallam T Abdel-Mobdy A Megahed Eissa A. Evaluation of oxidative stress, compositional and biochemical changes in milk and serum of cows with subclinical mastitis. Res Square. (2022) 3:1. 10.21203/rs.3.rs-1912881/v1

[44] Kapusta A Kuczyńska B Puppel K. Relationship between the degree of antioxidant protection and the level of malondialdehyde in high-performance Polish Holstein-Friesian cows in peak of lactation. PLoS ONE. (2018) 13:e0193512. 10.1371/journal.pone.0193512 29494660 PMC5832249

[45] Fernandes L Guimaraes I Noyes NR Caixeta LS Machado VS. Effect of subclinical mastitis detected in the first month of lactation on somatic cell count linear scores, milk yield, fertility, and culling of dairy cows in certified organic herds. J Dairy Sci. (2021) 104:2140–50. 10.3168/jds.2020-19153 33309348

[46] Halasa T Nielen M De Roos AP Van Hoorne R de Jong G Lam TJ. Production loss due to new subclinical mastitis in Dutch dairy cows estimated with a test-day model. J Dairy Sci. (2009) 92:599–606. 10.3168/jds.2008-1564 19164670

[47] Erwin RE Randolph HE. Influence of mastitis on properties of milk. XI Fat globule membrane J Dairy Sci. (1975) 58:9–12. 10.3168/jds.S0022-0302(75)84509-7 1112937

[48] Kumar S. Free Radicals and Antioxidants: Human and Food System. Adv Appl Sci Res. (2011) 2:129–35.

[49] Kalyanaraman B. Teaching the basics of redox biology to medical and graduate students: Oxidants, antioxidants and disease mechanisms. Redox Biol. (2013) 1:244–57. 10.1016/j.redox.2013.01.014 24024158 PMC3757692

[50] Aruoma OI. Nutrition and health aspects of free radicals and antioxidants. Food Chem Toxicol. (1994) 32:671–83. 10.1016/0278-6915(94)90011-6 8045480

[51] Esterbauer H. Cytotoxicity and genotoxicity of lipid-oxidation products. Am J Clin Nutr. (1993) 57:779–86. 10.1093/ajcn/57.5.779S 8475896

[52] Papastergiadis A Fatouh A Jacxsens L Lachat C Shrestha K Daelman J. Exposure assessment of Malondialdehyde, 4-Hydroxy-2-(E)-Nonenal and 4-Hydroxy-2-(E)-Hexenal through specific foods available in Belgium. Food Chem Toxicol. (2014) 73:51–8. 10.1016/j.fct.2014.06.030 25035169

[53] Leonarduzzi G Gargiulo S Gamba P Perrelli MG Castellano I Sapino A. Molecular signaling operated by a diet-compatible mixture of oxysterols in up-regulating CD36 receptor in CD68 positive cells. Mol Nutr Food Res. (2010) 54:S31–41. 10.1002/mnfr.200900493 20333725

[54] Staprans I Pan XM Rapp JH Feingold KR. Oxidized cholesterol in the diet accelerates the development of aortic atherosclerosis in cholesterol-fed rabbits. Arterioscler Thromb Vasc Biol. (1998) 18:977–83. 10.1161/01.atv.18.6.977 9633940

[55] Staprans I Pan XM Rapp JH Grunfeld C Feingold KR. Oxidized cholesterol in the diet accelerates the development of atherosclerosis in LDL receptor- and apolipoprotein E-deficient mice. Arterioscler Thromb Vasc Biol. (2000) 20:708–14. 10.1161/01.atv.20.3.708 10712395

[56] Staprãns I Rapp JH Pan XM Kim KY Feingold KR. Oxidized lipids in the diet are a source of oxidized lipid in chylomicrons of human serum. Arterioscler Thromb. (1994) 14:1900–5. 10.1161/01.atv.14.12.1900 7981177

[57] Staprans I Hardman DA Pan XM Feingold KR. Effect of oxidized lipids in the diet on oxidized lipid levels in postprandial serum chylomicrons of diabetic patients. Diabetes Care. (1999) 22:300–6. 10.2337/diacare.22.2.300 10333949

[58] Staprans I Pan XM Rapp JH Feingold KR. Oxidized cholesterol in the diet is a source of oxidized lipoproteins in human serum. J Lipid Res. (2003) 44:705–15. 10.1194/jlr.M200266-JLR200 12562864

[59] Li B Mo L Yang Y Zhang S Xu J Ge Y. Processing milk causes the formation of protein oxidation products which impair spatial learning and memory in rats. RSC Adv. (2019) 9:22161–75. 10.1039/c9ra03223a 35519476 PMC9066704

[60] Titov VY Kosenko OV Fisinin VI Klimov NT. Content of nitric oxide metabolites in cow milk in health and mastitis. Russ Agric Sci. (2010) 36:288–90. 10.3103/S1068367410040178

[61] Osman KM Hassan HM Ibrahim IM Mikhail MM. The impact of staphylococcal mastitis on the level of milk IL-6, lysozyme and nitric oxide. Comp Immunol Microbiol Infect Dis. (2010) 33:85–93. 10.1016/j.cimid.2008.08.009 18834632

[62] Moazeni M Heidari Z Golipour S Ghaisari L Sillanpää M Ebrahimi A. Dietary intake and health risk assessment of nitrate, nitrite, and nitrosamines: a Bayesian analysis and Monte Carlo simulation. Environ Sci Pollut Res Int. (2020) 27:45568–80. 10.1007/s11356-020-10494-9 32803593

[63] Sindelar JJ Milkowski AL. Human safety controversies surrounding nitrate and nitrite in the diet. Nitric Oxide. (2012) 26:259–66. 10.1016/j.niox.2012.03.011 22487433

[64] IARC Working Group on the Evaluation of Carcinogenic Risks to Humans. IARC monographs on the evaluation of carcinogenic risks to humans. Ingested nitrate and nitrite, and cyanobacterial peptide toxins. IARC Monogr Eval Carcinog Risks Hum. (2010) 94:v-vii, 1-412. PMC4781178 21141240

[65] Brenmoehl J Ohde D Wirthgen E Hoeflich A. Cytokines in milk and the role of TGF-beta. Best Pract Res Clin Endocrinol Metab. (2018) 32:47–56. 10.1016/j.beem.2018.01.006 29549959

[66] Dowlati Y Herrmann N Swardfager W Liu H Sham L Reim EK. A meta-analysis of cytokines in major depression. Biol Psychiatry. (2010) 67:446–57. 10.1016/j.biopsych.2009.09.033 20015486

[67] Swardfager W Lanctôt K Rothenburg L Wong A Cappell J Herrmann N. meta-analysis of cytokines in Alzheimer's disease. Biol Psychiatry. (2010) 68:930–41. 10.1016/j.biopsych.2010.06.012 20692646

[68] Locksley RM Killeen N Lenardo MJ. The TNF and TNF receptor superfamilies: integrating mammalian biology. Cell. (2001) 104:487–501. 10.1016/s0092-8674(01)00237-9 11239407

[69] Kokkonen H Söderström I Rocklöv J Hallmans G Lejon K Rantapää Dahlqvist S. Up-regulation of cytokines and chemokines predates the onset of rheumatoid arthritis. Arthritis Rheum. (2010) 62:383–91. 10.1002/art.27186 20112361

[70] Elenkov IJ Iezzoni DG Daly A Harris AG Chrousos GP. Cytokine dysregulation, inflammation and wellbeing. Neuroimmunomodulation. (2005) 12:255–69. 10.1159/000087104 16166805

[71] Hartwell D Levine J Fenton M Francis C Leslie C Beller D. Cytokine dysregulation and the initiation of systemic autoimmunity. Immunol Lett. (1994) 43:15–21. 10.1016/0165-2478(94)00144-8 7737685

[72] Ramachandran S Verma AK Dev K Goyal Y Bhatt D Alsahli MA. Role of Cytokines and Chemokines in NSCLC Immune Navigation and Proliferation. Oxid Med Cell Longev. (2021) 2021:5563746. 10.1155/2021/5563746 34336101 PMC8313354

[73] Landskron G. De la Fuente M, Thuwajit P, Thuwajit C, Hermoso MA. Chronic inflammation and cytokines in the tumor microenvironment. J Immunol Res. (2014) 2014:149185. 10.1155/2014/149185 24901008 PMC4036716

[74] D'Esposito V Di Tolla MF Lecce M Cavalli F Libutti M Misso S. Lifestyle and dietary habits affect plasma levels of specific cytokines in healthy subjects. Front Nutr. (2022) 9:913176. 10.3389/fnut.2022.913176 35811952 PMC9270017

[75] Smith CW Goldman AS. The cells of human colostrum. I In vitro studies of morphology and functions. Pediatr Res. (1968) 2:103–9. 10.1203/00006450-196803000-00005 4173318

[76] Bannerman DD. Pathogen-dependent induction of cytokines and other soluble inflammatory mediators during intramammary infection of dairy cows. J Anim Sci. (2009) 87:10–25. 10.2527/jas.2008-1187 18708595

[77] Wellnitz O Baumert A Saudenowa M Bruckmaier RM. Immune response of bovine milk somatic cells to endotoxin in healthy quarters with normal and very low cell counts. J Dairy Res. (2010) 77:452–9. 10.1017/S0022029910000348 20822558

[78] Kiełbasa A Gadzała-Kopciuch R Buszewski B. Cytokines-biogenesis and their role in human breast milk and determination. Int J Mol Sci. (2021) 22:6238. 10.3390/ijms22126238 34207900 PMC8229712

[79] Georgiades JA Fleischmann WR Jr. Oral application of cytokines. Biotherapy. (1996) 8:205–12. 10.1007/BF01877206 8813332 PMC7088415

[80] Solomon A Richey T Murphy CL Weiss DT Wall JS Westermark GT. Amyloidogenic potential of foie gras. Proc Natl Acad Sci U S A. (2007) 104:10998–1001. 10.1073/pnas.0700848104 17578924 PMC1894569

[81] Gruys E. Protein folding pathology in domestic animals. J Zhejiang Univ Sci. (2004) 5:1226–38. 10.1631/jzus.2004.1226 15362194 PMC1388739

[82] Zhang H Sawashita J Fu X Korenaga T Yan J Mori M. Transmissibility of mouse AApoAII amyloid fibrils: inactivation by physical and chemical methods. FASEB J. (2006) 20:1012–4. 10.1096/fj.05-4890fje 16549653

[83] Lundmark K Westermark GT Nyström S Murphy CL Solomon A Westermark P. Transmissibility of systemic amyloidosis by a prion-like mechanism. Proc Natl Acad Sci U S A. (2002) 99:6979–84. 10.1073/pnas.092205999 12011456 PMC124514

[84] Greger M. Amyloid fibrils: Potential food safety implications. Int. J. Food Safety Nutr. Public Health. (2008) 1:103–115. 10.1504/ijfsnph.2008.023011

[85] De Buck M Gouwy M Wang JM Van Snick J Proost P Struyf S. The cytokine-serum amyloid A-chemokine network. Cytokine Growth Factor Rev. (2016) 30:55–69. 10.1016/j.cytogfr.2015.12.010 26794452 PMC7512008

[86] Nazifi S Khoshvaghti A Gheisari HR. Evaluation of serum and milk amyloid A in some inflammatory diseases of cattle. Iran J Vet Res. (2008) 9:222–6.

[87] Szczubiał M Dabrowski R Kankofer M Bochniarz M Komar M. Concentration of serum amyloid A and ceruloplasmin activity in milk from cows with subclinical mastitis caused by different pathogens. Pol J Vet Sci. (2012) 15:291–6. 10.2478/v10181-011-0149-x 22844707

[88] Vasil M Elečko J Farkašová Z Zigo F. Diagnostic importance of the concentration of milk amyloid A in quarter milk samples from dairy cows with mastitis. Acta Vet Brno. (2012) 81:133–8. 10.2754/avb20128102013321134311

[89] Thorn DC. Amyloid Fibril Formation by Bovine Milk α*s*_2_*- and* κ*-casein, and Its Inhibition by the Molecular Chaperones* α*s1- and* β*-casein*. PhD Thesis University of Adelaide, School of Chemistry and Physics, Adelaide, South Australia, Australia (2010).33545456

[90] Auldist MJ Coats S Sutherland BJ Mayes JJ McDowell GH Rogers GL. Effects of somatic cell count and stage of lactation on raw milk composition and the yield and quality of Cheddar cheese. J Dairy Res. (1996) 63:269–80. 10.1017/s0022029900031769 8861348

[91] Rogers SA Slattery SL Mitchell GE Hirst PA Grieve PA. The relationship between somatic cell count, composition and manufacturing properties of bulk milk III Individual proteins. Aust J Dairy Technol. (1989) 44:49–52.

[92] Forsbäck L Lindmark-Månsson H Andrén A Svennersten-Sjaunja K. Evaluation of quality changes in udder quarter milk from cows with low-to-moderate somatic cell counts. Animal. (2010) 4:617–26. 10.1017/S1751731109991467 22444049

[93] Randolph HE Erwin RE Richter RL. Influence of mastitis on properties of milk. VII distribution of milk proteins. J Dairy Sci. (1974) 57:15–8. 10.3168/jds.S0022-0302(74)84824-1 4810480

[94] Schaar J Funke H. Effect of subclinical mastitis on milk plasminogen and plasmin compared with that on sodium, antitrypsin and N-acetyl-beta-D-glucosaminidase. J Dairy Res. (1986) 53:515–28. 10.1017/s0022029900033045 2947939

[95] Le Roux Y Colin O Laurent F. Proteolysis in samples of quarter milk with varying somatic cell counts. 1 Comparison of some indicators of endogenous proteolysis in milk. J Dairy Sci. (1995) 78:1289–97. 10.3168/jds.S0022-0302(95)76749-2 7673517

[96] Leitner G Merin U Silanikove N. Changes in milk composition as affected by subclinical mastitis in goats. J Dairy Sci. (2004) 87:1719–26. 10.3168/jds.S0022-0302(04)73325-1 15453484

[97] Leitner G Chaffer M Shamay A Shapiro F Merin U Ezra E. Changes in milk composition as affected by subclinical mastitis in sheep. J Dairy Sci. (2004) 87:46–52. 10.3168/jds.S0022-0302(04)73140-9 14765809

[98] Heegaard CW Christensen T Rasmussen MD Benfeldt C Jensen NE Sejrsen K. Plasminogen activators in bovine milk during mastitis, an inflammatory disease. Fibrinolysis. (1994) 8:20–30. 10.1016/0268-9499(94)90028-0

[99] Modh RH Islam MM Nauriyal DS Modi RJ Wadhwani. Study on pH and somatic cell count in milk of sub-clinical mastitic cows in association with udder and teat shape. Indian J Anim Prod Mgmt. (2018) 34:75–9.

[100] Politis I Ng Kwai Hang KF Giroux RN. Environmental factors affecting plasmin activity in milk. J Dairy Sci. (1989) 72:1713–8. 10.3168/jds.S0022-0302(89)79286-9 2528574

[101] Mangione PP Verona G Corazza A Marcoux J Canetti D Giorgetti S. Plasminogen activation triggers transthyretin amyloidogenesis *in vitro*. J Biol Chem. (2018) 293:14192–9. 10.1074/jbc.RA118.003990 30018138 PMC6139548

[102] Slamova I Adib R Ellmerich S Golos MR Gilbertson JA Botcher N. Plasmin activity promotes amyloid deposition in a transgenic model of human transthyretin amyloidosis. Nat Commun. (2021) 12:7112. 10.1038/s41467-021-27416-z 34876572 PMC8651690

[103] Rising A Gherardi P Chen G Johansson J Oskarsson ME Westermark GT. AA amyloid in human food chain is a possible biohazard. Sci Rep. (2021) 11:21069. 10.1038/s41598-021-00588-w 34702933 PMC8548551

[104] Rahman MM Pires RS Herneke A Gowda V Langton M Biverstål H. Food protein-derived amyloids do not accelerate amyloid β aggregation. Sci Rep. (2023) 13:985. 10.1038/s41598-023-28147-5 36720893 PMC9889329

[105] Liu J Wang J Xu W Zeng L Wang C An Y. Amyloid fibril formation by casein and fatty acid composition in breast milk of mastitis patients. J Food Biochem. (2022) 46:e14183. 10.1111/jfbc.14183 35383958

[106] Sgorlon S Fanzago M Guiatti D Gabai G Stradaioli G Stefanon B. Factors affecting milk cortisol in mid lactating dairy cows. BMC Vet Res. (2015) 11:259. 10.1186/s12917-015-0572-9 26459289 PMC4603817

[107] Robertson D Robertson RM. “Chapter 133 – Fludrocortisone - Clinical Pharmacology,” in *Primer on the Autonomic Nervous System* (Third Edition), ed. D Robertson, I Biaggioni, G Burnstock, PA Low, JFR Paton (Academic Press). (2012) 635–637. Available online at: https://www.sciencedirect.com/topics/neuroscience/fludrocortisone/ (accessed May 3, 2023).

[108] Winder CB Sargeant JM Kelton DF Leblanc SJ Duffield TF Glanville J. Comparative efficacy of blanket versus selective dry-cow therapy: a systematic review and pairwise meta-analysis. Anim Health Res Rev. (2019) 20:217–28. 10.1017/S1466252319000306 32081118

[109] Niemi RE Hovinen M Vilar MJ Simojoki H Rajala-Schultz PJ. Dry cow therapy and early lactation udder health problems—Associations and risk factors. Prev Vet Med. (2021) 188:105268. 10.1016/j.prevetmed.2021.105268 33530013

[110] Guth M Smedzik-Ambrozy K Czyzewski B Stepień S. The economic sustainability of farms under common agricultural policy in the European Union countries. Agriculture. (2020) 10:34. 10.3390/agriculture10020034

[111] Barkema HW Schukken YH Zadoks RN. Invited Review: The role of cow, pathogen, and treatment regimen in the therapeutic success of bovine Staphylococcus aureus mastitis. J Dairy Sci. (2006) 89:1877–95. 10.3168/jds.S0022-0302(06)72256-1 16702252

[112] Park S Ronholm J. Staphylococcus aureus in agriculture: lessons in evolution from a multispecies pathogen. Clin Microbiol Rev. (2021) 34:e00182–20. 10.1128/CMR.00182-20 33568553 PMC7950364

